# Green Synthesis of Copper and Copper Oxide Nanoparticles From Brown Algae Turbinaria Species' Aqueous Extract and Its Antibacterial Properties

**DOI:** 10.7759/cureus.57366

**Published:** 2024-04-01

**Authors:** San Chitta Raj Raja Rajamanikkam, Geetha Anbalagan, Balachandran Subramanian, Vasugi Suresh, Pitchiah Sivaperumal

**Affiliations:** 1 Department of Physiology, Saveetha Dental College and Hospitals, Saveetha Institute of Medical and Technical Sciences (SIMATS) Saveetha University, Chennai, IND; 2 Department of Prosthodontics, Saveetha Dental College and Hospitals, Saveetha Institute of Medical and Technical Sciences (SIMATS) Saveetha University, Chennai, IND

**Keywords:** copper oxide nanoparticles, scanning electron microscope, antibacterial activity, characterization techniques, turbinaria algae

## Abstract

Background

Copper and copper oxide nanoparticles synthesized by green methods have attracted considerable attention due to their environmentally friendly properties and potential applications. Green synthesis involves non-hazardous and sustainable techniques used in the production of a wide range of substances, including nanoparticles, pharmaceuticals, and chemicals. These methods often use different organisms, including bacteria, fungi, algae, and plants, each offering different advantages in terms of simplicity, cost-effectiveness, and environmental sustainability. The environmentally friendly nature of these green synthesis methods responds to the growing need for sustainable nanotechnologies. Brown algae have gained popularity due to their distinct morphological characteristics and diverse biochemical composition. This research focuses on the process of synthesizing copper and copper oxide nanoparticles from the brown algae *Turbinaria*. It emphasizes the natural ability of the bioactive compounds contained in the algae extract to reduce and stabilize the nanoparticles. The green synthesis of copper and copper oxide nanoparticles from brown algae has demonstrated a wide range of applications, including antibacterial activity.

Materials and methods

Fresh *Turbinaria* algae were collected from marine environments to ensure that they were free of contaminants. The algae underwent a purification process to remove impurities and were dried. An aqueous extract was prepared by pulverizing the dried algae and mixing them with distilled water. A copper salt solution utilizing copper nitrate was prepared. The algae extract was mixed with the copper salt solution. There are bioactive compounds in the algae extract that help reduce copper ions, which makes copper and copper oxide nanoparticles come together. The reaction mixture was incubated in a controlled environment to facilitate the growth and enhance the stability of the nanoparticles. To separate the nanoparticles from the reaction mixture, centrifugation was employed, or filtration was done with Whatman filter paper (Merck, Burlington, MA). The nanoparticles were dried to yield a stable powder.

Results

Copper and copper oxide nanoparticles derived from brown algae extract showed antibacterial effects against *Streptococcus mutans, Klebsiella sp., *and *Staphylococcus mutans*. The scanning electron microscopy (SEM) analysis verified the irregular shape and elemental content of the synthesized copper and copper oxide nanoparticles. The X-ray diffraction (XRD) analysis indicated that the synthesized nanoparticles exhibited a crystallinity nature and were composed of a mixture of copper and copper oxide species, namely face-centered cubic and monoclinic structures. The transmission electron microscopy (TEM) images showed copper and copper oxide nanoparticles that were evenly distributed and had a rectangular shape. They exhibited substantial antimicrobial activity against both Gram-positive and Gram-negative bacteria.

Conclusions

This study enhances the field of green synthesis techniques by showcasing the adaptability of *Turbinaria *brown algae to synthesize copper and copper oxide nanoparticles. It underscores the potential advantages of these nanoparticles in terms of their antibacterial properties.

## Introduction

Nanoparticles can be synthesized using diverse techniques such as chemical reduction [1], pulse laser ablation [2], hydrothermal synthesis [3], and green synthesis methods [4]. Nanoparticles have been extensively studied in the last decade due to their distinctive characteristics, which include electronic, optical, magnetic, physical, chemical, mechanical, and thermal properties. The process of chemically synthesizing nanoparticles can potentially result in toxicity, which gives rise to concerns regarding their effects on human health and the environment [5, 6].

Green synthesis refers to environmentally friendly techniques used to synthesize various materials, chemicals, or nanoparticles. The green synthesis method utilizes leaf extracts [7] and microorganisms (fungi, bacteria, and algae) [8] to reduce toxicity. The green synthesis of nanoparticles follows sustainable practices and offers an eco-friendly alternative to traditional methods [9]. Metal oxide nanoparticles synthesized using plant extract play a crucial role in nanotechnology due to their wide application in various industries and pharmaceuticals. They serve as disinfectants, catalysts, fillers, and antibiotics targeting microorganisms. The biosynthesis of metal oxide nanoparticles has various reactions against microorganisms based on their size [10].

*Turbinaria *brown algae are marine macroalgae with a distinct fan-shaped or turbine-like appearance. *Turbinaria* species are frequently found in tropical and southeast coastal areas. *Turbinaria *species exhibit a range of colors, ranging from olive brown to dark brown, due to the presence of pigments such as chlorophylls and fucoxanthin. The *Turbinaria *genus of brown algae has become notable for its distinct morphological characteristics and abundant biochemical composition. *Turbinaria* species of brown algae have shown great potential as suitable candidates for the environmentally friendly synthesis of nanoparticles due to their naturally occurring bioactive compounds [11,12].

A number of researchers are interested in making copper oxide nanoparticles because they have unique physicochemical properties and can be used in many different fields [13]. *Turbinaria *brown algae, known for its high concentration of bioactive compounds, is an effective reducing and stabilizing agent in the nanoparticle synthesis process. The aqueous extract of *Turbinaria*, which consists of polysaccharides and polyphenols, enables the reduction of copper ions to produce stable copper oxide nanoparticles. Copper oxide nanoparticles exhibit strong antibacterial efficacy against various microorganisms, encompassing bacteria, fungi, and viruses. They have the ability to disturb the cell membranes, hinder enzyme activity, and provoke oxidative stress in bacterial cells, resulting in their destruction [14-17].

This study elucidates the synthesis of copper and copper oxide nanoparticles using *Turbinaria *brown algae, a highly promising, environmentally friendly method that has a wide range of applications in the fields of biomedicine and the environment. *Turbinaria*-derived copper and copper oxide nanoparticles possess favorable biocompatibility and inherent characteristics, rendering them highly promising for applications such as drug delivery systems, imaging agents, and other therapeutic uses. Copper and copper oxide nanoparticles possess the functionality of antibacterial activity [18-21], rendering them highly versatile materials with potential applications in diverse domains such as healthcare, environmental remediation, and renewable energy production. The process is explained using different analytical techniques, including X-ray diffraction analysis (XRD), transmission electron microscopy (TEM), scanning electron microscopy (SEM), and its antibacterial activity.

## Materials and methods

Origin of the sample

The *Turbinaria* brown algae used in this study was collected from the Tuticorin region of Tamil Nadu, India. The brown algae, *Turbinaria*, was washed and sterilized using distilled water to remove any surface contaminants. A temperature-controlled oven was used to dehydrate the sample, ensuring that the temperatures were kept below 60°C. The dried sample was pulverized into granules using an electric blender. The powdered samples were sealed tightly and stored out of direct sunlight in order to preserve them for later use [22]. 

Preparation of *Turbinaria* brown algae extract

*Turbinaria *brown algae powder (5 g) was added to a conical flask containing 50 mL of ethanol. The mixture was vigorously stirred for two hours at 60°C. It was allowed to cool and filtered through Whatman filter paper (Merck, Burlington, MA) to obtain the solution.

Synthesis of copper oxide nanoparticles

**Figure 1:**
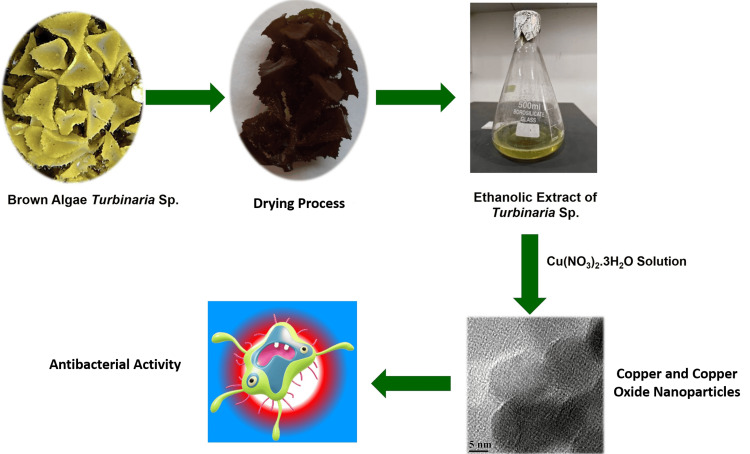
A symmetric diagram illustrating the green synthesis of copper and copper oxide nanoparticles from the brown algae Turbinaria Cu(NO_3_)_2_.3H_2_O: copper (II) nitrate trihydrate

The synthesis of copper and copper oxide nanoparticles was a simple, cost-effective, green synthesis approach. Five grams of copper(II)nitrate trihydrate (Cu(NO_3_)_2_.3H_2_O) were added to a conical flask containing 50 mL of ethanol. The resulting mixture was then stirred for two hours at 60°C, and cooled.* Turbinaria* brown algae extract (20 mL) was gradually added to a 50 mL copper (II) nitrate trihydrate (Cu(NO_3_)_2_.3H_2_O) solution. An orbital shaker was used to stir the resulting mixture at a speed of 280 rpm for 24 hours at 60°C. The solution was subjected to an orbital shaker at room temperature to reduce the amount of copper nanoparticles. The solution's copper ion concentration was periodically monitored using a UV spectrophotometer. The copper and copper oxide nanoparticles underwent purification through a sequence of centrifugation procedures, with each procedure lasting 10 minutes and operating at a speed of 8,000 rpm. The water-suspended nanoparticles were subjected to a 24-hour vacuum drying process to eliminate moisture from the nanoparticles. Subsequently, this extract underwent testing to determine its antibacterial properties. The copper precursor solution is then combined with the algae extract, which is rich in bioactive chemicals. Polysaccharides and polyphenols, two bioactive components found in algae extract, have a lowering effect. They reduce copper ions to copper and copper oxide nanoparticles by donating electrons to the ions. Some algae chemicals, such as those with hydroxyl (OH-) groups, have functional groups that help with this reduction process. Not only do the bioactive chemicals help with copper ion reduction, but they also stabilize copper and copper oxide nanoparticles, keep them from clustering together, and make the colloidal solution stable (Figure 1).

Antibacterial activity

The disk diffusion method was employed to assess the antibacterial efficacy of copper and copper oxide nanoparticles from brown algae extract. The copper and copper oxide nanoparticles (50 mg) were diluted in 2.5 mL of ethanol, sterilized using a Millipore filter (0.22 mm, Merck), and then applied onto sterile filter paper discs (8 mm in diameter) to achieve a final concentration. Ten milliliters of Mueller-Hilton agar media were put into sterilized Petri dishes as a bottom layer. Filter paper discs impregnated with copper and copper oxide nanoparticles at a concentration of 10 mg/ml were placed on the surface of Mueller-Hilton agar plates. Filter paper discs containing 20 µg of tetracycline were used as a positive control. The plates were refrigerated at 5°C for two hours to allow copper and copper oxide nanoparticles to diffuse, then incubated at 35°C for 24 hours. The inhibitory zones were measured using a Vernier caliper, recorded, and interpreted as proof of antibacterial activity.

## Results

X-ray diffraction

**Figure 2:**
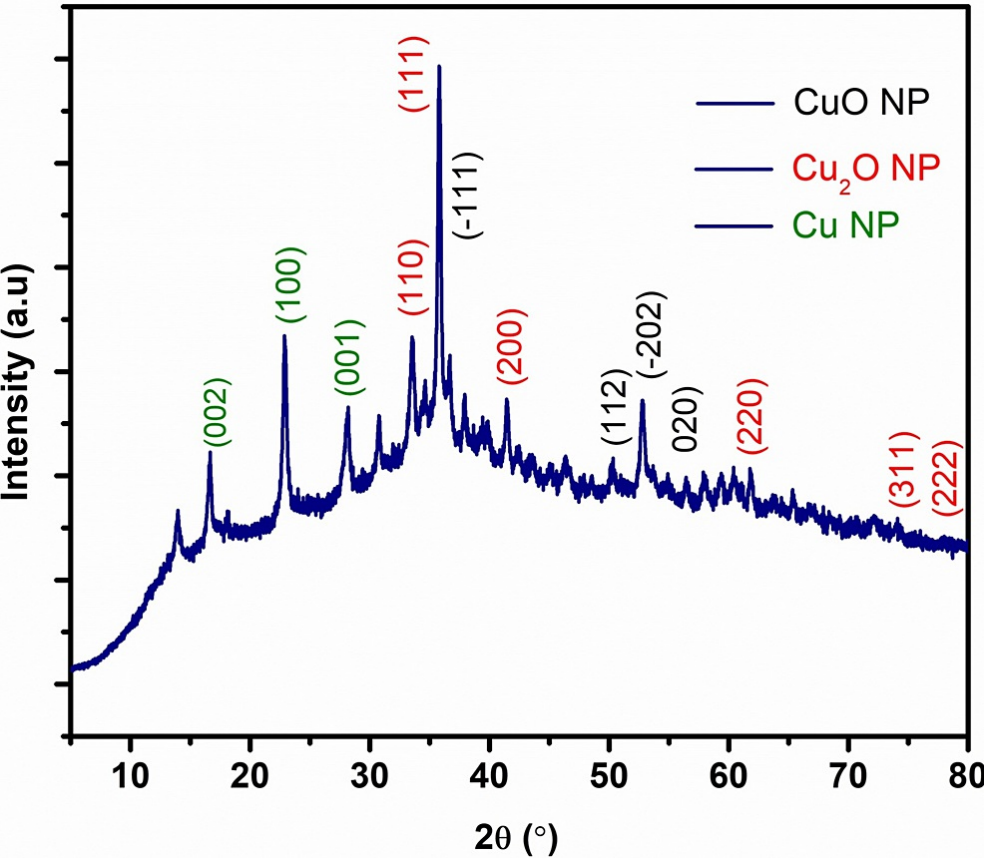
The XRD analysis of copper and copper oxide nanoparticles derived from brown algae XRD: X-ray diffraction; Cu NP: copper nanoparticle; CuO NP, Cu_2_O NP: copper oxide nanoparticle

Copper and copper oxide nanoparticles had a prominent Bragg diffraction peak at 36.3° (2 theta), along with smaller but distinct peaks. The peaks represent distinct crystallographic planes of the face-centered cubic structure of copper oxide nanoparticles, specifically the (110), (111), (200), (220), (311), and (222) planes, according to the Joint Committee on Powder Diffraction Standards (JCPDS) card no. 05-667. As particle size decreases, XRD reflection peaks broaden due to lattice flaws, stacking faults, and other causes, which is a frequent phenomenon in nanoparticles that is dependent on size. The copper oxide nanoparticles have a monoclinic structure with specific crystallographic planes: (-111), (-202), and (020). No additional diffraction peaks indicating impurities were seen in the XRD spectra of copper nanoparticles and copper oxide nanoparticles. The copper nanoparticles are responsible for the diffraction planes of (002), (100), and (001) (Figure 2).

Scanning electron microscopy

**Figure 3:**
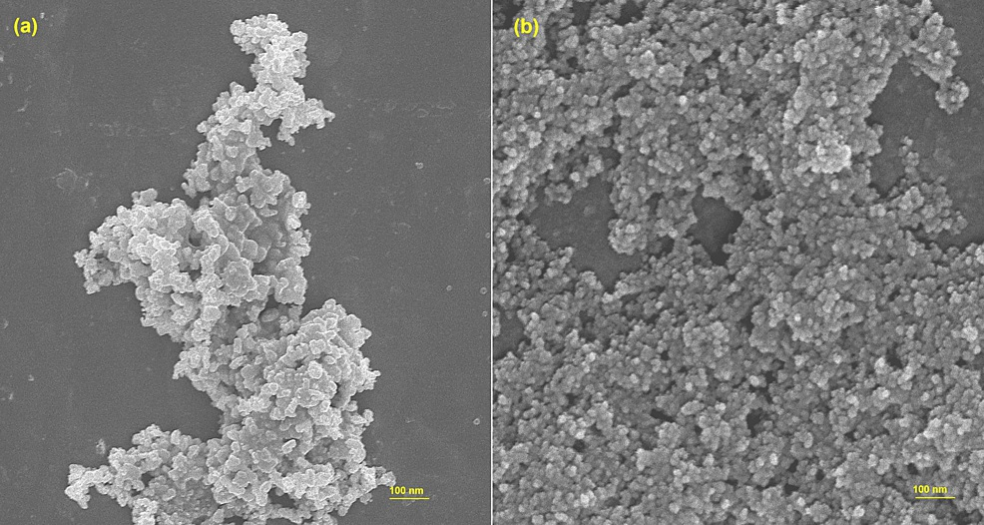
The SEM image of copper and copper oxide nanoparticles derived from brown algae (a and b) at 100 nm SEM: scanning electron microscopy

Figures 3a-3b illustrate the SEM images of copper and copper oxide nanoparticles derived from brown algae at 7.5K and 5K magnification, respectively. The scanning electron micrographic images show that the copper and copper oxide nanoparticles are tightly packed together and have a particle-like structure. As a result of the drying process, the particles attached to nearby particles, resulting in a cluster-like shape. Particles were uniform in shape and often less than 20 nm in size.

Transmission electron microscopy

**Figure 4:**
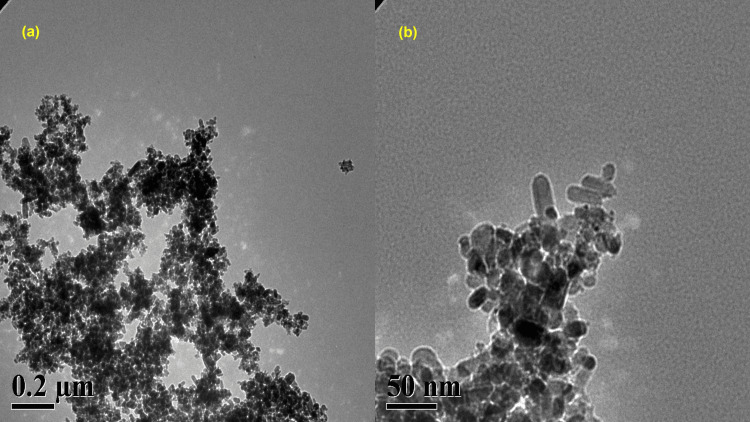
The TEM image of copper and copper oxide nanoparticles derived from brown algae; (a) 0.2 µm and (b) 50 nm TEM: transmission electron microscopy

The TEM images in Figure 4 clearly show the uniform dispersion of copper and copper oxide nanoparticles with a size of 18 ± 2 nm. The nanoparticles displayed a consistent structure and a near rectangle shape. The consistent arrangement of the nanoparticles in the high-magnification TEM image suggests that the copper and copper oxide nanoparticles were equally distributed. The TEM image revealed well-dispersed and rectangular copper and copper oxide nanoparticles.

Antibacterial activity

**Figure 5:**
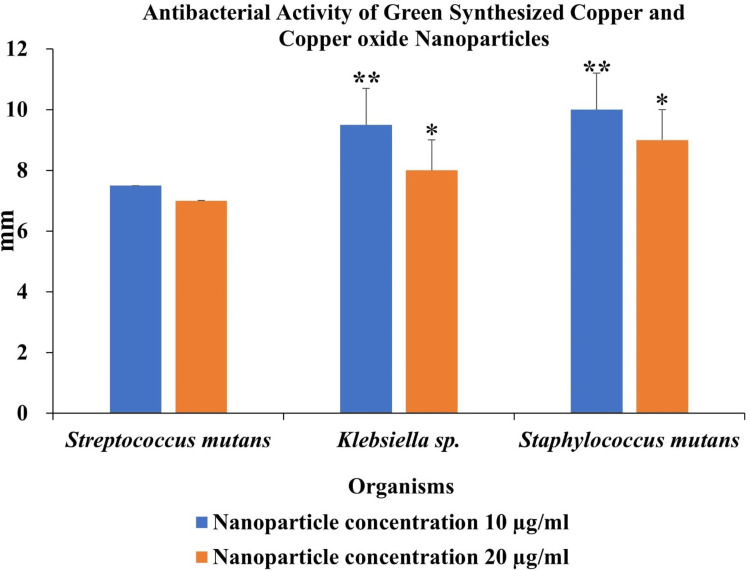
Antibacterial activity of green synthesized copper and copper oxide nanoparticles against Streptococcus mutans, Klebsiella sp., and Staphylococcus mutans

The antibacterial effectiveness of green synthesized copper and copper oxide nanoparticles against Gram-positive bacteria (*Streptococcus mutans, Staphylococcus mutans*)and Gram-negative bacteria(*Klebsiella sp*.) was investigated. The copper and copper oxide nanoparticles demonstrated efficacy in inhibiting the growth of microorganisms. Two different concentrations of copper and copper oxide nanoparticles derived from brown algae extract (10, 20 µg/ml) were used to determine the zone of inhibition against organisms. The copper and copper oxide nanoparticles synthesized from brown algae extract exhibited a moderate zone of inhibition against gram-positive bacteria and gram-negative bacteria. Tetracycline was used as a positive control (20 µg/disc). The antibacterial activities of tetracycline were 22 mm, 22 mm, and 16 mm for *Streptococcus mutans,* *Klebsiella sp.,* and *Staphylococcus mutans,* respectively. In the case of copper and copper oxide nanoparticles, the antibacterial activity is presented in Figure 5. The zones of inhibition were 7.5, 9.5, and 10 mm for *Streptococcus mutans,* *Klebsiella sp.,* and *Staphylococcus mutans*, respectively, at 10 µg/ml.

## Discussion

Copper nanoparticles have antimicrobial action, and it is shown that the bactericidal effect is mainly caused by the release of copper ions when nanoparticles come into contact with bacterial cells. Inducing oxidative stress and interfering with vital cellular functions, these ions damage cell membrane integrity and kill bacteria. The work highlights the complex antibacterial effect of copper nanoparticles, which might have several uses in the fight against bacterial diseases. The results help uncover better ways to fight microbes and create new materials that are resistant to bacteria by illuminating the processes at work. This study opens the door to new antibacterial agents and materials by shedding light on the potential uses of copper nanoparticles in environmental and medicinal contexts [23]. Recent studies have documented the synthesis of copper oxide nanoparticles through various green synthesis and biologically based techniques [3, 10, 24]. The Cu^2+^ and silver ions (Ag^+^) were thoroughly investigated for their ability to penetrate bacterial cell membranes and affect enzyme function. Indirect impacts on the surrounding charge environment also influence the efficacy of nanoparticulate metals against microorganisms [24]. Surface functionalization is crucial for improving antibacterial activity since surface-modified copper nanoparticles outperform their unmodified counterparts in terms of antibacterial efficiency. According to Fayaz et al., this discovery points to a potential way forward for creating more effective antibacterial materials [25].

The main focus of this study is on making, improving, and analyzing synthesized copper and copper oxide nanoparticles from *Turbinaria *brown algae extract. The study was conducted in a regulated setting and under close supervision. The primary benefit of utilizing the green pathway for the synthesis of copper and copper oxide nanoparticles is the achievement of stabilization [26]. The concentration of phytochemicals in the extract is crucial for the formation and stabilization of copper and copper oxide nanoparticles. By elevating the concentration of the extract, the rate of reduction of Cu^2+^ ions will be accelerated, leading to a decrease in the size of copper oxide nanoparticles [27]. Intrigued about the antibacterial efficacy of copper oxide nanoparticles against both Gram-positive and Gram-negative bacteria, the subject was thoroughly investigated. The work showcased the wide-ranging antibacterial potential of copper oxide nanoparticles by showing how they significantly suppress bacterial growth [28]. Copper oxide nanoparticles have antibacterial properties that might help fight bacterial infections. Copper oxide nanoparticles caused oxidative stress in bacterial cells, which damaged DNA and cell membranes. They shed light on the molecular pathways that are responsible for the antibacterial activities of copper oxide nanoparticles and offered important insights into how these nanoparticles work. To tackle the urgent problem of multidrug-resistant strains, the research highlighted the possible function of copper oxide nanoparticles in overcoming bacterial resistance [29]. Nevertheless, any cytotoxicity concerns related to nanoparticle usage must be carefully considered. When deciding how to use copper oxide nanoparticles, it is important to consider the antibacterial activity of these particles in relation to any potential harmful effects, their biocompatibility and cytotoxicity, and the foundation for future research on the antibacterial activity of copper oxide nanoparticles, which could lead to the development of safe and effective antibacterial agents [30].

Limitation

Due to the presence of a number of bioactive compounds, the reduction of nanoparticles is not complete and forms a mixture of copper and copper oxide nanoparticles. Green synthesis methods may introduce impurities that arise from *Turbinaria* brown algae used in the synthesis process. It can be difficult to ensure the resultant nanoparticles are highly pure, and extra purification procedures might be needed. Green synthesis methods may have limited control over the size and shape of the resulting nanoparticles. The lack of control can have an impact on the properties of nanoparticles and their applications. Green synthesized copper and copper oxide nanoparticles might not be as stable or have a longer shelf life than nanoparticles made with conventional chemical methods. This instability can restrict their capacity for storage and practical usefulness.

## Conclusions

The study on *Turbinaria* brown algae-derived copper and copper oxide nanoparticles and their antibacterial properties revealed that these nanoparticles are efficient in combating bacteria. X-ray diffraction analysis revealed that the synthesized nanoparticles exhibited a high degree of crystallinity and consisted of a combination of copper and copper oxide nanoparticles, namely face-centered cubic and monoclinic structures. Scanning electron microscopy images indicated that both the copper and copper oxide nanoparticles exhibited a structure characterized by the agglomeration of particles. Transmission electron microscopy images revealed the presence of well-dispersed copper and copper oxide nanoparticles that were designed using a green method. These nanoparticles exhibited a rectangular configuration. The findings demonstrate the efficacy of copper and copper oxide nanoparticles as antibacterial agents.
